# Does Publication History Predict Future Publication Output in Orthopaedics?

**DOI:** 10.7759/cureus.15273

**Published:** 2021-05-27

**Authors:** Madison L Goss, Sarah McNutt, Jesse E Bible

**Affiliations:** 1 Orthopaedics and Rehabilitation, Penn State Health Milton S. Hershey Medical Center, Hershey, USA; 2 Neurosurgery, Penn State Health Milton S. Hershey Medical Center, Hershey, USA

**Keywords:** academic research, orthopaedic research, orthopaedic surgery, scholarly productivity, research publications, publication trends, research and publication

## Abstract

Background

The number of publications is widely used as a measure of academic productivity in the field of orthopaedics. How “productive” a physician is has a great influence on consideration for employment, compensation, and promotions. Predictors of potential high-output researchers would be of value to the orthopaedic department and university leadership for new faculty evaluation.

Methods

The study population included orthopaedic faculty from the top 10 orthopaedic institutions in the United States. Their names and the number of publications at each point in their training (medical school, residency, and fellowship) and early career (first five and 10 years following fellowship) along with a total number of publications to date were collected by using PubMed.

Results

Strong correlations were seen between publications during total training and publications output in the first five years following fellowship (*r_s_*=0.717, P<0.0001). However, no significant correlations were found comparing publications during each stage of training and the first 10 years following fellowship. A moderate positive correlation was found when comparing publications during medical school and residency output (*r_s_*=0.401, P<0.0001).

Conclusions

The data presented here may be utilized by department chairs during the evaluation of faculty and candidates to not interpret the number of publications during training and early career as a gauge of research interest and potential for future publications. Program directors may also use the only moderate correlation between publications in medical school and residency when evaluating applications as support of a more holistic review of applicants to determine research interest.

## Introduction

The importance of publications early in one's career in determining successful applications for medical school, residency, and fellowship has been well documented [1]. Publication frequency, referred by some as academic productivity, can also serve as a determinant for physician compensation along with academic rank later in one’s career [1]. Peer-reviewed publications have become an essential component of not only promotion but also maintaining a position within academics. Within the field of orthopaedics, the literature has shown that faculty with 10 plus years of work as an attending had significantly more publications than those under 10 years [2].

Considering the significant impact of a physician’s publication history on their academic career within orthopaedics, research regarding relationships between publication record during training and early career with future academic productivity is of particular interest to not only orthopaedic leadership but also recruitment and promotion committees. Many times, an orthopaedic surgeon’s prior publication history is translated into their academic potential later in their career. The purpose of this study is to assess if prior publication output during training and early years as an attending correlates with future publication output within orthopaedics.

## Materials and methods

The top 10 academic institutions in the United States for orthopaedics were identified using the US News Health Report Rankings in 2018 [3]. The online directories were searched for each institution and the names of all orthopaedic surgeons were collected. Publicly available information from hospital websites and curricula vitae were used to obtain graduation years for medical school, residency, and any fellowship(s) the physician completed. PubMed was used to identify the number of peer-reviewed publications from each physician at each stage in their training (medical school, residency, fellowship), the number of publications in the five and 10 years following fellowship, and the total number of publications prior to and including the year 2018. When available, the date of acceptance of publication was used rather than the date of publication to attempt to account for delays in publication. H-indexes for each physician were obtained from Scopus.

Physicians with incomplete data, such as missing graduation years during training, were omitted from data analysis. Spearman’s rank correlation coefficients (*r_s_*, SPSS 26.0; IBM, Chicago, IL, USA) were used to assess the relationships between the number of publications at different points in training and the early years in practice. Strength of the correlation was described using the following guide for the absolute value for *r_s_*: 0.00-0.19 “very weak”, 0.20-0.39 “weak”, 0.40-0.59 “moderate”, 0.60-0.79 “strong”, 0.80-1.0 “very strong”. Physicians with multiple fellowships were included in each of their respective fellowships for analysis.

## Results

Complete data were able to be obtained for 355 physicians employed by the top 10 academic institutions for orthopaedic surgery (Hospital for Special Surgery, Mayo Clinic, Cleveland Clinic, Rothman Institute, Rush University, Massachusetts General, University of California San Francisco, New York University Langone, Cedars-Sinai Medical Center, and Johns Hopkins University). The sample included 327 (92%) males and 28 (8%) females.

The majority were listed as assistant professor (46%), followed by associate professor (32%), and professor (22%). Years in practice, the total number of publications, and H-indexes all showed positive trends with increasing academic rank (Table 1).

**Table 1:** Average years in practice, publications, and H-indexes between academic ranks. The average years in practice, number of publications, and H-indexes increased with increasing academic rank from assistant professor to associate professor and finally to professor.

Academic Rank	Years in Practice	# of Publications	H-index
Assistant Professor	15.9+/-10.9	14.1+/-15.5	6.1+/-6.2
Associate Professor	24.3+/-11.8	46.9+/-44.9	15.7+/-13.9
Professor	26.1+/-9.5	56.4+/-67.4	20.2+/-15.5

The average number of publications during medical school was 0.3+/-3.3 (Table 2). The average number of publications during residency and fellowship were 1.6+/-5.0 and 1.2+/-3.1, respectively. The average for total number of publications during training (medical school, residency, and fellowship) was 3.0+/-8.6. In the first five years in practice, the average number of publications was 4.5+/-10.2 and in the 10 years following fellowship was 33.8+/-52.5. The average for the total number of publications to date was 36.7+/-51.7. The average H-index was calculated to be 12.3+/-14.4.

A comparison of academic productivity between the orthopaedic subspecialties including the number of publications and per cent of physicians with no publications at each stage in training is displayed in Table 2.

**Table 2:** Publication data at different timepoints by orthopaedic subspecialty. The average number of publications and the per cent of physicians with no publications at different timepoints for each of the seven subspecialties in orthopaedics: trauma, joints, sports, shoulder/elbow, spine, hand, paediatrics, foot and ankle, and oncology. The average number of publications as an attending physician per year for each subspecialty is reported to account for differences in career lengths.

Orthopaedic Subspecialty			Medical School		Residency		Fellowship		First 5 years in Practice		First 10 years in Practice		Current Total				
	# of Attendings	Years in Practice	# of Publications	% with No Publications	# of Publications	% with No Publications	# of Publications	% with No Publications	# of Publications	% with No Publications	# of Publications	% with No Publications	# of Publications	H-index	# of Publications/Yrs in Practice	# of Publications as Attending/Yrs in Practice	
																	Trauma	37	20.9+/-11.4	0.2+/-0.6	86%	0.8+/-2.1	76%	0.8+/-1.8	70%	5.7+/-10.1	49%	47.8+/-62.5	3%	45.3+/-60.5	15.7+/-12.4	2.4+/-2.9	2.2+/-2.8	
Joints	62	18.2+/-11.6	2.1+/-10.7	84%	2.6+/-6.2	65%	1.8+/-3.5	63%	7.9+/-15.4	35%	46.3+/-53.2	6%	52.4+/-52.9	16.1+/-14.9	4.2+/-5.3	3.2+/-3.8	
Sports	88	19.4+/-10.3	0.2+/-0.6	89%	1.4+/-4.3	69%	0.9+/-1.9	67%	5.6+/-14.2	49%	36.5+/-52.0	15%	39.3+/-53.9	11.3+/-13.0	2.4+/-3.5	2.0+/-3.1	
Shoulder/Elbow	14	19.9+/-10.7	0.1+/-0.3	93%	2.6+/-5.2	64%	0.7+/-1.5	71%	4.1+/-8.0	50%	52.2+/-51.2	0%	49.1+/-47.8	22.5+/-19.3	2.7+/-2.4	2.3+/-1.9	
Spine	48	18.7+/-9.2	0.4+/-1.8	92%	1.3+/-3.1	75%	0.9+/-2.2	77%	3.9+/-7.5	52%	30.2+/-57.8	15%	42.5+/-58.6	13.2+/-14.8	2.7+/-3.3	2.3+/-3.0	
Hand	43	20.0+/-13.1	0.1+/-0.5	93%	2.7+/-5.6	63%	1.2+/-2.2	65%	6.0+/-11.4	42%	48.3+/-68.7	9%	45.7+/-62.4	13.3+/-14.8	2.8+/-3.3	2.2+/-2.8	
Paediatrics	28	17.3+/-9.5	0.1+/-0.3	89%	1.7+/-4.7	79%	0.8+/-2.1	82%	4.8+/-9.7	39%	20.9+/-34.5	11%	22.6+/-35.0	8.5+/-10.6	2.1+/-3.6	1.4+/-2.1	
Foot & Ankle	21	16.6+/-10.5	0.2+/-0.8	90%	1.0+/-2.0	67%	1.1+/-2.8	67%	4.7+/-8.9	43%	22.9+/-31.7	14%	22.9+/-27.7	7.2+/-8.4	1.7+/-1.9	1.4+/-1.7	
Oncology	11	19.1+/-12.7	0.1+/-0.3	91%	0.8+/-1.2	55%	1.0+/-1.9	64%	3.7+/-4.6	45%	25.4+/-24.9	9%	22.5+/-21.5	15.3+/-10.8	1.5+/-1.2	1.2+/-0.9	
Total	352	19.8+/-11.3	0.5+/-4.4	98%	1.6+/-4.3	77%	1.1+/-2.4	77%	5.3+/-11.5	51%	35.8+/-52.1	13%	39.6+/-51.8	13.4+/-14.0	2.6+/-3.6	2.1+/-2.9	

Spearman rank coefficients showed statistically significant correlations between the number of publications in medical school, residency, fellowship, and the first five years in practice (Table 3). Strong correlations were seen between publications during total training and publications output in the first five years in practice (*r_s_*=0.717, P<0.0001). However, no significant correlations were found between the number of publications at any stage in training and the number of publications produced in the first 10 years following fellowship. Furthermore, only a weak positive correlation was observed between the number of publications during the first five and first 10 years following fellowship (*r_s_*=0.274, P<0.0001).

**Table 3:** Correlations between publication history and future output. Spearman rank correlation coefficients with corresponding p-values for the number of publications between different stages in training and early career.

Does publication history predict future publication output?				
	Residency	Fellowship	First 5 yrs in Practice	First 10 yrs in Practice
Medical School (MS)	0.401 (<0.0001)	0.303 (<0.0001)	0.256 (<0.0001)	-0.053 (0.365)
Residency (R)	--	0.706 (<0.0001)	0.666 (<0.0001)	0.036 (0.540)
Fellowship (F)		--	0.632 (<0.0001)	0.062 (0.318)
Total Training (MS, R, and F)			0.717 (<0.0001)	0.075 (0.195)
First 5 yrs in Practice			--	0.274 (<0.0001)

A moderate positive correlation was found when comparing publications during medical school and residency output (*r_s_*=0.401, P<0.0001) and a strong correlation was seen with publications during residency and publications during fellowship (*r_s_*=0.706, P<0.0001).

## Discussion

The output of peer-reviewed publications, commonly referred to as academic productivity, is viewed as an academic currency (along with research funding) by departmental and university leadership. Although an isolated publication number may not accurately represent academic output, it is used as a global assessment for when promotion, dedicated academic time and funding, and institutional value come into question. 

A strong significant correlation was found between publication output during training with output during the first five years following completion of the fellowship. This finding could suggest that having a previous foundation in research can help an attending be productive earlier in their career. This foundation could come in the form of training/experience in efficient data collection, statistics, or manuscript preparation, along with already established academic collaborations.

However, no significant correlation was seen between productivity during training with productivity in the 10 years following fellowship. Furthermore, only a weak correlation was seen between publication output during the first five years and the first 10 years. These results can be important for orthopaedic leadership to take note of. A new attending may not have a strong prior research foundation or may hold other time consuming academic/clinical duties that could lead to lower publication output during their early career. However, this should not always be viewed as them not having an “interest” in research, seeing as they still have the potential to become a high-output researcher later in their career. A major component in consideration for promotion is current research productivity and the potential for future productivity, as measured by the number of peer-reviewed publications. This information may be used by department chairs when evaluating the portfolios of assistant and associate professors for under consideration for promotion. A physician’s lack of early publications during training years should not be used as an indicator of future research productivity potential. This information may also be used when hiring new faculty that may not have produced many publications during their years of training, but demonstrate many other characteristics that align with the institution’s values and criteria for junior faculty.

In addition to using by department chairs when evaluating faculty, orthopaedic residency program directors may use this data when evaluating medical students’ applications. In recent years, the number of publications has become increasingly important for medical students as a determinate of successfully matching into an orthopaedic residency program. When selecting applicants to interview, 66% of orthopaedic surgery program directors cited "demonstrated involvement and interest in research" as a factor in applicant selection [4]. In 2018, the average number of abstracts, presentations, and publications listed on a successfully matched medical student’s application in orthopaedics was 11.9, which is almost double the average number listed on unmatched medical students’ applications for orthopaedics [5]. Here we demonstrate that the correlation between publications during medical school and residency was only moderate at 0.401 for physicians who went on to become faculty at highly ranked institutions. This data support decreasing the emphasis on the number of publications and advocate for a more holistic review of each applicant in consideration for interviews invites and the creation of rank lists.

This study was not without limitations; the first being the selection bias of only including orthopaedic surgeons employed by the top 10 institutions. While highly ranked institutions tend to conduct and publish more research, this still represents a limitation in the utility of subspecialty references at other institutions. It was not feasible to collect data for the orthopaedic faculty at every academic centre, so we chose to focus on these institutions where we predicted this type of data would be of greatest utility to department chairs. The other main limitation of this study was that not every journal lists the date of acceptance along with the date of publication, so publication date had to be used when the acceptance date was not available. With the time from initial submission to publication estimated to be 36 weeks, the significant correlations seen between sequential stages in training, such as residency and fellowship, may be influenced by a lag in publication times [6]. Lastly, while the number of publications has been widely used as a measure of academic productivity, the quality and impact of each publication should also be considered. While we did not assess the quality of each individual publication, the use of PubMed helped to ensure only peer-reviewed articles within the field of biomedical sciences and life sciences were counted.

## Conclusions

This paper provides some useful reference data for residency directors, department chairs by using data obtained from the top 10 orthopaedic institutions. Here we demonstrated that while there is some correlation between the number of publications during training with the number of publications during the first five years following fellowship, this relationship does not hold true for number of publications during the first 10 years following fellowship. Therefore, department chairs should not view the number of publications as a reflection of a new attending's research interest. This information may also be utilized by residency program directors while evaluating medical students’ applications as a reminder to look beyond the number of publications when gauging a potential resident's research interest.
